# Literary Notes

**Published:** 1901-04

**Authors:** 


### LITERARY NOTES.

MESSRS. LEA BROTHERS & CO., of Philadelphia, announce for early issue A Practical Treatise on the Blood and Its Diseases, for practitioners, laboratory workers and students, by James Ewing, M.D., professor of pathology in Cornell University Medical College, New York. This will be a handsome octavo volume of about 450 pages amply illustrated with plates and engravings.

In view of the recent rapid advances in the knowledge of the pathology of the blood, and the numerous and practical applications of this knowledge in clinical diagnosis this book, representing authoritatively as it does the most modern discoveries and achievements, will no doubt meet with a warm welcome.

---

THE New York University, beginning with February, 1901, publishes a bi-weekly bulletin, which takes the place of the annual circulars heretofore issued. One of the new features is the *Bulletin of the Medical Sciences*, which is to be devoted to reports of research and discovery in medicine, is to contain no advertisements, is not issued for profit, but, on the contrary, will cost the University money to publish. The engraving of a single page in the second number is to cost $300.

---

MESSRS. BATTLE & CO., St. Louis, Mo., have issued Chart No. 3 of the series illustrating fractures of long bones. This relates to fracture of the femur above the condyle, and, like the others, is printed in color. It presents graphically this important injury, showing the relation of the broken bone to the soft parts. It will be sent on application to the publishers.

---

THE *University Medical Magazine*, Philadelphia, will hereafter be known as the *Medical Bulletin of the University of Pennsylvania*. It will contain no advertising matter and its form has been changed into a size which we abominate. Why medical publications continue to issue in a size so inconvenient that they neither are read nor preserved, we are at a loss to understand. The policy and scope of the publication remains practically unchanged; it continues to be the official journal of the medical department and departments allied to medicine, of the University of Pennsylvania Medical Society and of the William Pepper laboratory of clinical medicine. Its contents will consist of original articles, complete and carefully prepared digests of the most recent literature upon a subject of practical or scientific importance, alumni notes and book reviews.

